# Impact of Pre-operative Aerobic Exercise on Cardiometabolic Health and Quality of Life in Patients Undergoing Bariatric Surgery

**DOI:** 10.3389/fphys.2020.01018

**Published:** 2020-08-26

**Authors:** Nicole M. Gilbertson, Natalie Z. M. Eichner, Mahnoor Khurshid, Elizabeth A. Rexrode, Sibylle Kranz, Arthur Weltman, Peter T. Hallowell, Steven K. Malin

**Affiliations:** ^1^Department of Kinesiology, University of Virginia, Charlottesville, VA, United States; ^2^Department of Surgery, University of Virginia, Charlottesville, VA, United States; ^3^Department of Medicine, University of Virginia, Charlottesville, VA, United States; ^4^Robert M. Berne Cardiovascular Research Center, University of Virginia, Charlottesville, VA, United States

**Keywords:** arterial stiffness, C-reactive protein, cytokeratin 18, physical activity, dietary intake

## Abstract

**Objective:**

Examine the effect of aerobic exercise (EX) combined with standard medical care (SC) (EX + SC) compared to SC alone on cardiometabolic health and quality of life in relation to surgical outcomes.

**Methods:**

Patients receiving bariatric surgery were match-paired to 30 days of pre-operative SC (*n* = 7, 1 male, 39.0 ± 5.3 years, body mass index 46.4 ± 3.0 kg/m^2^; low calorie diet) or EX + SC (*n* = 7, 0 males, 45.6 ± 4.8 years, body mass index 43.9 ± 4.2 kg/m^2^; walking 30 min/day, 5 days/week, 65–85% HR_*peak*_). Body mass, waist circumference, cardiorespiratory fitness (VO_2_peak), high sensitivity C-reactive protein (hs-CRP), cytokeratin 18 (CK18), weight related quality of life (QoL), and a 120 min mixed meal tolerance test (MMTT) was performed to assess arterial stiffness via augmentation index normalized to a heart rate of 75 beats per minute (AIx@75), whole-body insulin sensitivity, and glucose total area under the curve (tAUC) pre- and post-intervention (∼2 days prior to surgery). Length of hospital stay (admission to discharge) was recorded.

**Results:**

EX + SC had a greater effect for decreased intake of total calories (*P* = 0.14; ES = 0.86) compared to SC, but no change in body weight or waist circumference was observed in either group. EX + SC had a greater effect for increased VO_2_peak (*P* = 0.24; ES = 0.91) and decreased hs-CRP (*P* = 0.31; ES = 0.69) compared to SC. EX + SC reduced circulating CK18 (*P* = 0.05; ES = 3.05) and improved QoL (*P* = 0.02) compared to SC. Although EX + SC had no statistical effect on arterial stiffness compared to SC, we observed a modest effect size for AIx@75 tAUC (*P* = 0.36; ES = 0.52). EX + SC had a significantly shorter length of hospital stay (*P* = 0.05; ES = 1.38) than SC, and a shorter length of hospital stay was associated with decreased sugar intake (*r* = 0.55, *P* = 0.04). Decreased AIx@75 tAUC significantly correlated with improved whole-body insulin sensitivity (*r* = −0.59, *P* = 0.03) and glucose tAUC (*r* = 0.57, *P* = 0.04).

**Conclusion:**

EX with SC for 30 days prior to bariatric surgery may be important for cardiometabolic health, quality of life, and surgical outcomes in the bariatric patient.

## Introduction

Morbid obesity rates continue to rise in the United States (Sturm and Hattori, 2013) due to, in part, excess caloric intake and physical inactivity (Hills et al., 2007). The risk of cardiometabolic disease mortality increases 7% for every two additional years lived with obesity (Reis et al., 2013). Obesity is also related to decreased overall quality of life (QoL) (Faulconbridge et al., 2013; Ul-Haq et al., 2013). This is problematic because reduced QoL propagates a state of over-eating and decreased physical activity that exacerbates obesity related comorbidities (Rosemann et al., 2008; Faulconbridge et al., 2013). Roux-en-Y Gastric Bypass (RYGB) and Sleeve Gastrectomy (SG) are the two most common forms of bariatric surgery in the United States and they elicit an approximate 50% reduction in excess body weight and significantly improve cardiometabolic health and QoL (Schauer et al., 2012; Li et al., 2014). Despite these benefits, the number of intra- and post-operative complications, length of operating time, as well as length of hospital stay varies among patients (Fernandez et al., 2004; Steinbrook, 2004). Pre-operative cardiometabolic disease risk factors (e.g., fasting glucose, blood pressure, and lipids) (Purnell et al., 2014), liver health (Colles et al., 2006; Edholm et al., 2011), and high sensitivity C-reactive protein (hs-CRP) have been linked to length of hospital stay and surgical complications in bariatric patients (Purnell et al., 2014; Goh et al., 2018). Further, a peak cardiorespiratory fitness (VO_2_peak) < 15.8 mL/kg/min is associated with longer operating time, greater estimated blood loss during surgery, and more frequent surgical complications (McCullough et al., 2006). Thus, improving cardiometabolic health prior to bariatric surgery may be a reasonable target of interventions to enhance surgical outcomes.

Current standard medical practice prior to bariatric surgery emphasizes a low-calorie diet (LCD) to reduce liver size and body weight for improved surgical outcomes (Nguyen et al., 2005; Van Nieuwenhove et al., 2011). A plethora of evidence indicates the importance of physical activity for overall cardiometabolic health and well-being (Penedo and Dahn, 2005; Shiroma Eric and Lee, 2010). However, standard medical care does not currently include a physical activity or exercise component for the bariatric patient. Furthermore, <10% of bariatric patients meet current physical activity recommendations (Bond et al., 2010), albeit approximately 40% of bariatric patients feel more ready to exercise 2 weeks prior to surgery (Bond et al., 2010). Interestingly, we recently showed that adding aerobic exercise to standard care (EX + SC) prior to bariatric surgery reduced length of hospital stay compared to standard care (SC) alone, and reduced length of hospital stay correlated with increased VO_2_peak (Gilbertson et al., 2020). This suggests that increasing cardiorespiratory fitness prior to bariatric surgery by adding aerobic exercise to a LCD can improve surgical outcomes. Combining aerobic exercise with a LCD has also been shown to lower arterial stiffness and traditional cardiometabolic disease risk factors (e.g., blood pressure, fasting glucose, and lipids) more than either intervention alone in some (Azadbakht et al., 2005; Anderssen et al., 2007; Blumenthal et al., 2010) but not all studies (Yassine et al., 2009; Weiss, 2016). To date, no study has evaluated the effect of adding a pre-operative EX intervention to SC compared to SC alone on cardiometabolic health in relation to surgical outcomes. Therefore, the purpose of the present study was to evaluate the effect of pre-operative SC versus EX + SC on cardiometabolic health and QoL in relation to surgical outcomes in patients receiving bariatric surgery. We tested the hypothesis that EX + SC would have greater improvements in pre-operative cardiometabolic health and QoL compared to SC alone and this would relate to improved surgical outcomes as well as physical activity and dietary intake in patients receiving bariatric surgery.

## Materials and Methods

### Participants

Patients approved for bariatric surgery at the University of Virginia were recruited between 2015 and 2018. Participants with obesity (BMI of 30–70 kg/m^2^) were included for participation in the study if they were 18–70 years old, undergoing their first RYGB or SG procedure, not pregnant or lactating, or not taking medications known to alter body weight. Participants were excluded if they were physically active (>60 min/wk of exercise), diagnosed with insulin dependent diabetes, had a history of cardiovascular disease, or diagnosed with cancer in the past 5 years. Physicals and physician clearance for participation in the study was completed by one investigator (P.T.H.). All outcomes were assessed pre-intervention and post-intervention (i.e., ∼2 days prior to surgery). After pre-intervention testing participants were match paired to 30 days of pre-operative EX + SC or SC based on body mass index (BMI), sex, race, and surgery type (RYGB or SG). One investigator (P.T.H.) completed all RYGB or SG surgical procedures and was blinded to the patient’s treatment group. Length of hospital stay was defined as the time of hospital admission to discharge, and surgical residents were responsible for hospital discharge and had no knowledge of the patient’s participation in the present study. Operating time was defined as time of incision to close. These data, along with demographic characteristics were previously published (Gilbertson et al., 2020) but are included herein for ease of the reader. All participants provided written and verbal informed consent as approved by our Institutional Review Board.

### Body Composition and Cardiorespiratory Fitness

Body weight was measured to the nearest 0.01 kg on a digital scale and height was measured with a stadiometer to assess BMI. Fat mass and fat free mass (FFM) were measured using air displacement plethysmography (Bod Pod, Concord, CA, United States). Waist circumference was measured 2 cm above the umbilicus using a flexible tape measure. VO_2_peak was determined using a treadmill exercise test with indirect calorimetry (Carefusion, Vmax CART, Yorba Linda, CA, United States). Participants self-selected a speed and grade was increased 2.5% every 2 min until volitional exhaustion. The highest heart rate value achieved during the VO_2_peak test was recorded as heart rate peak (HR_*peak*_) and used for the exercise prescription of EX + SC.

### Cardiometabolic Risk Factors

Metabolic control was implemented for 48 h prior to testing and included avoidance of alcohol, caffeine, dietary supplements, medication, and non-exercise physical activity for 24 h prior to testing. The last exercise bout was performed approximately 24 h before post-intervention testing. Participants were admitted to the Clinical Research Unit at approximately 8:00 a.m. following an overnight fast. Participants laid supine undisturbed for about 5 min to determine resting heart rate and blood pressure (BP) with Dinamap (CARESCAPE V100 monitor, GE Healthcare), which was averaged over three measurements for data analysis. Mean arterial pressure (MAP) = [((2^∗^diastolic) + systolic)/3] was calculated. An intravenous catheter was then placed in an antecubital vein. Fasting blood was collected to measure glucose, insulin, triglycerides (TG), high-density lipoproteins (HDL), low-density lipoproteins (LDL), total cholesterol, high sensitivity CRP (hs-CRP), and cytokeratin 18 (CK18), a marker of hepatocyte apoptosis and liver health. A mixed meal tolerance test (MMTT) was then administered in which participants consumed 4 fl. oz. of an Ensure Plus (Abbott Park, Illinois) shake (CHO 25 g, fat 5.5 g, and protein 6.5 g), and circulating glucose and insulin was also determined every 30 min up to 120 min after consumption of the mixed meal. The Matsuda Index was used to estimate whole-body insulin sensitivity (Matsuda and DeFronzo, 1999). Indirect calorimetry (Carefusion, Vmax CART, Yorba Linda, CA, United States) with a ventilated hood was utilized to assess respiratory exchange ratio (RER) at 0, 60, and 120 min of the MMTT. Metabolic flexibility was determined by subtracting fasting RER from the average of post-prandial RER as previously reported (Gilbertson et al., 2018).

### Pulse Wave Analysis

Fasting and postprandial pulse pressure (PP), augmentation pressure (AP), and augmentation index corrected to a heart rate of 75 bpm using the manufacturer’s software (AIx@75) was measured by applanation tonometry using the SphygmoCor^®^ system (AtCor Medical, Itasca, IL, United States) at 0, 60, and 120 min of the MMTT. Pulse wave analysis measures were recorded at each time point in a temperature-controlled room while patients rested quietly in the supine position. AIx@75 total area under the curve (tAUC) was determined using the trapezoid method (Allison et al., 1995).

### Quality of Life

The Laval questionnaire was used to assess weight related QoL as it has been validated in participants with morbid obesity and is sensitive to treatment-induced changes (Therrien et al., 2011). The questionnaire includes 44 questions, each expressed on a 7-point Likert scale, assessing 6 domains including symptoms, sexual life, activity/mobility, personal hygiene/clothing, emotions, and social interactions. Total score was calculated by adding all six domains together and dividing by the total possible points (Baillot et al., 2013; Accardi et al., 2017). Scores were transformed into a percentage, and a higher score is indicative of higher QoL.

### Non-exercise and Total Physical Activity

Accelerometers (Actigraph GT3X+, Pensacola, FL, United States) were used to assess non-exercise physical activity for 1 week prior to the mixed meal tolerance test (MMTT) at pre- and post-intervention testing. The average wear time per day and the total number of days the device was worn at each time point is reported. Freedson VM3 (’11) is an energy expenditure algorithm that was used to determine the frequency of sedentary bouts per day as well as percent of wear time spent sedentary, in light physical activity, or in moderate to vigorous physical activity (MVPA) per day (Sasaki et al., 2011). Individuals in EX + SC were told to remove the accelerometer during their EX bout(s) which is outlined below. Total physical activity was determined by adding exercise time recorded by the A300 Polar fitness and activity trackers to accelerometer wear time.

### Standard Medical Care (SC)

Participants met with registered dieticians, attended an education session with a registered nurse, and cleared for bariatric surgery by a psychologist and the surgical team prior to study enrollment. Patients attended a pre-operative visit approximately 40 days prior to bariatric surgery. For 2 weeks prior to surgery, patients were instructed by registered dieticians to consume a meal replacement shake for breakfast and lunch, snacks of raw vegetables, a dinner composed of 4 oz. of lean protein and steamed vegetables, and sugar-free beverages.

### Exercise (EX)

Participants in EX + SC also completed at home walking for 30 min/day, 5 days/week, 65–85% HR_*peak*_ for 30 days. Exercise adherence was monitored by the research team using an exercise diary and an A300 Polar fitness and activity tracker (Kempele, Finland). The Polar fitness monitor tracked the duration, heart rate, and calories expended per exercise session. The research team also conducted weekly check-ins by texting, emails, and/or phone calls with study participants to promote adherence.

### Dietary Analysis

Three-day food logs, including two weekdays and one weekend day, were used to assess *ad libitum* food intake. Participants were provided with reference guides that displayed serving sizes of beverages and food commonly consumed. They were also given detailed instructions for recording food and beverages consumed over the intervention. Intake was analyzed with the Food Processor Nutrition Analysis Software (ESHA Research, Version 11.1, Salem, OR, United States).

### Biochemical Analysis

Aprotinin was added to the EDTA vacutainer tube prior to blood collection for insulin. Blood was collected in EDTA and SST vacutainer tubes as well as lithium heparin microtainer bullet tubes and immediately placed on ice. Lithium heparin microtainer bullet tubes were then immediately centrifuged at 13,300 rpm for 60 s, and the YSI 2300 StatPlus Glucose Analyzer system (Yellow Springs, OH, United States) was used to determine plasma glucose. SST tubes rested for 30 min prior to processing. All EDTA and SST vacutainers were centrifuged for 10 min at 4°C and 3,000 rpm, and samples was aliquoted to cryotubes and stored at −80°C until later analysis. Samples were batch analyzed in duplicate to minimize inter-assay variability. The University of Virginia’s Health System Medical Laboratories determined HDL and total cholesterol as well as TG by colorimetric assays from serum collected in SST tubes. LDL cholesterol was calculated from the Friedewald equation (Friedewald et al., 1972). Plasma collected in EDTA tubes was used for the determination of insulin (Alpco, Salem, NH, United States), hs-CRP (Millipore, Billerica, MA, United States), and CK18 (M30 apoptosense ELISA, Diapharma Group, West Chester, OH, United States) by using enzyme-linked immunosorbent assays, and the intra-assay and inter-assay coefficient of variation was <7.9 and <9.9%, respectively, for these analytes.

### Statistical Analysis

It was determined that 5 obese adults would be needed to show the effect of short-term EX on AIx@75 (delta of 782, SD of 397 with 80% power and alpha of 0.05) (Eichner et al., 2019) and insulin sensitivity (delta of 0.5, SD of 0.9 with 80% power and alpha of 0.05) (Kelly et al., 2012). Data were analyzed using SPSS Version 26 (IBM Analytics, Armonk, NY, United States). Normality was assessed using Shapiro–Wilk tests. Baseline differences and surgical outcomes were analyzed using independent samples *t*-tests. A non-parametric Wilcoxon Signed Rank test was performed to determine differences from pre- to post-intervention in weight related QoL, and Mann–Whitney *U* Test evaluated differences between groups in the score change from pre- to post-intervention. All other outcomes were analyzed using a 2 × 2 repeated measure analysis of variance (ANOVA). Cohen’s *d* effect sizes (ES) were also calculated on the interaction of treatments for physiological outcomes, and relevance was interpreted as small (*d* = 0.2), medium (*d* = 0.5), or large (*d* = 0.8). Pearson’s correlation was used to assess associations. Body composition, VO_2_peak, whole-body insulin sensitivity, and metabolic flexibility was previously reported (Gilbertson et al., 2020), but these data are used herein to determine associations between metabolic health and cardiovascular disease risk factors, dietary intake, as well as physical activity. Significance was set at *P* ≤ 0.05. Data are presented as mean ± SEM.

## Results

### Patient Characteristics

There was no difference between SC (*n* = 7) and EX + SC (*n* = 7) in age (SC 39.0 ± 5.3 vs. EX + SC 45.6 ± 4.8 years), sex (SC, *n* = 6 females vs. EX + SC, *n* = 7 females), race/ethnicity (SC, *n* = 5 Caucasians, 2 African Americans vs. EX + SC, *n* = 5 Caucasians, *n* = 1 African American, *n* = 1 Pacific Islander), or type of surgical procedure (SC, *n* = 3 RYGB, *n* = 4 SG vs. EX + SC, *n* = 3 RYGB, *n* = 4 SG) (all, *P* > 0.37). Although body weight (SC −0.6 ± 1.6 vs. EX + SC −0.5 ± 0.7 kg) and waist circumference (SC −1.1 ± 0.6 vs. EX + SC −0.8 ± 1.4 cm) was unchanged with SC and EX + SC, FFM decreased with both interventions (*P* = 0.05) and SC (−1.5 ± 0.5kg) had a greater decrease in FFM than EX + SC (−0.6 ± 0.8kg) (*P* = 0.39; ES = 0.48). There was a medium effect size (*P* = 0.36; ES = 0.52) for an increase in fat mass with SC (0.9 ± 1.0kg) compared to a slight reduction with EX + SC (−0.3 ± 0.8kg). EX + SC had a large effect size for increased VO_2_peak relative to body weight (mL/kg/min) (*P* = 0.24; ES = 0.91; SC −1.2 ± 0.9 vs. EX + SC 0.5 ± 1.0 mL/kg/min) compared to SC, albeit not statistically significant.

### Dietary Intake

Both interventions decreased fat (g; *P* = 0.003), saturated fat (g; *P* = 0.02), trans fat (g; *P* = 0.05), cholesterol (mg; *P* = 0.009), and fiber (g; *P* = 0.003) over the study (Table 1). EX + SC had a greater effect for decreased intake of total calories (Table 1; *P* = 0.14; ES = 0.86) compared to SC. EX + SC also had a greater effect for decreased intake of CHO (g; *P* = 0.07; ES = 1.06), sugar (g; *P* = 0.19; ES = 0.75), and unsaturated fat (g; *P* = 0.008; ES = 1.71) compared to increased intake with SC (Table 1).

**TABLE 1:** Effect of standard care (SC) and aerobic exercise combined with standard care (EX + SC) on dietary intake.

	SC		EX + SC		ANOVA (*P*-value)		Effect size
	PRE	Δ	PRE	Δ	*T*	*G* × *T*	Cohen’s *d*
Calories	1921 ± 273	−95 ± 332	1986 ± 232	−747 ± 234	0.06	0.14	0.86
Carbohydrates							
Grams	182.9 ± 29.7	19.8 ± 53.0	228.1 ± 30.6	−108.5 ± 37.0	0.20	0.07	1.06
% total kcal	37.7 ± 2.3	6.5 ± 5.7	45.5 ± 2.2	−8.2 ± 5.4	0.30	0.38	1.00
Fiber (g)	13.1 ± 2.0	−2.3 ± 2.5	20.4 ± 2.7	−11.0 ± 2.6	0.001	0.34	1.31
Sugar (g)	72.2 ± 14.2	12.0 ± 22.5	71.4 ± 6.9	−22.8 ± 10.4	0.67	0.19	0.75
Protein							
grams	112.0 ± 24.7	0.3 ± 17.4	82.0 ± 10.6	−13.6 ± 11.8	0.54	0.52	0.35
% total kcal	22.8 ± 2.6	−0.1 ± 2.7	16.7 ± 1.2	11.7 ± 5.2	0.47	0.22	1.07
Fat							
Grams	82.9 ± 10.7	−20.5 ± 10.8	87.7 ± 9.7	−38.2 ± 11.2	0.003	0.28	0.61
% total kcal	39.8 ± 2.2	−7.3 ± 3.4	40.0 ± 1.3	−8.0 ± 4.8	0.02	0.91	0.10
Saturated fat (g) | |	26.0 ± 3.0	−7.9 ± 3.1	24.8 ± 3.0	−8.3 ± 5.1	0.02	0.95	0.04
Unsaturated fat (g)	25.3 ± 5.9	0.5 ± 2.8	29.0 ± 4.6	−17.5 ± 4.9	0.01	0.008	1.71
*Trans* fat (g)	0.80 ± 0.21	−0.4 ± 0.2	0.52 ± 0.22	−0.2 ± 0.2	0.05	0.65	0.26
Cholesterol (mg)	426.0 ± 98.1	−130.4 ± 33.3	307.0 ± 70.2	−135.7 ± 78.3	0.009	0.95	0.03
Water (g)	2195 ± 463	128.3 ± 815.2	1539 ± 296	31.2 ± 202.7	0.85	0.91	0.06

**Data are means ± SEM. Fiber, % carbohydrates of total kcal, and % protein of total kcal were covaried for baseline values due to differences between groups. Change from pre- to post-intervention (Δ); grams (g); time (T; pre vs. post-intervention); group × time (G × T; SC vs. EX + SC)*.*

### Exercise and Non-exercise Physical Activity

EX + SC participants completed 18.4 ± 3.0 exercise sessions for 37.3 ± 2.6 min/session at moderate to vigorous intensity (75.7 ± 0.02% HR_*peak*_) which equated to 248.8 ± 24.0 kcal/session. There was no difference in days of accelerometer wear between SC and EX + SC at baseline (SC 6.2 ± 0.6 vs. EX + SC 6.3 ± 0.2 days; *P* = 0.53) nor was there a difference between groups after treatment (SC 5.0 ± 0.3 vs. EX + SC 5.8 ± 0.5 days; *P* = 0.32; ES = 0.61). Both treatments had the same frequency of sedentary bouts per day at baseline (SC 9.4 ± 1.4 vs. EX + SC 15.6 ± 2.4 bouts; *P* = 0.24) and after treatment (SC 5.2 ± 0.5 vs. EX + SC 13.4 ± 3.6 bouts; *P* = 0.72; ES = 0.34). Non-exercise sedentary time appeared to increase with EX + SC from baseline (SC 30.1 ± 5.2 vs. EX + SC 38.5 ± 2.3%) to post-intervention (SC 29.0 ± 5.5 vs. EX + SC 41.5 ± 5.8%; *P* = 0.09). This observation remained for percent of total time spent sedentary (including exercise within the day) from baseline (SC 30.1 ± 5.2 vs. EX + SC 38.5 ± 2.3%) to post-intervention (SC 29.0 ± 5.5 vs. EX + SC 39.0 ± 5.2%; *P* = 0.08). SC and EX + SC had no changes in percent of non-exercise time spent in light physical activity or MVPA (data not shown). However, EX + SC (baseline 6.9 ± 0.8 vs. post-intervention 11.2 ± 1.1%) spent significantly more of their total time in MVPA compared to SC (baseline 4.4 ± 0.8 vs. post-intervention 3.9 ± 0.9%) during the study (*P* = 0.004; ES = 3.01).

### Blood Pressure and Pulse Wave Analysis

**FIGURE 1:**
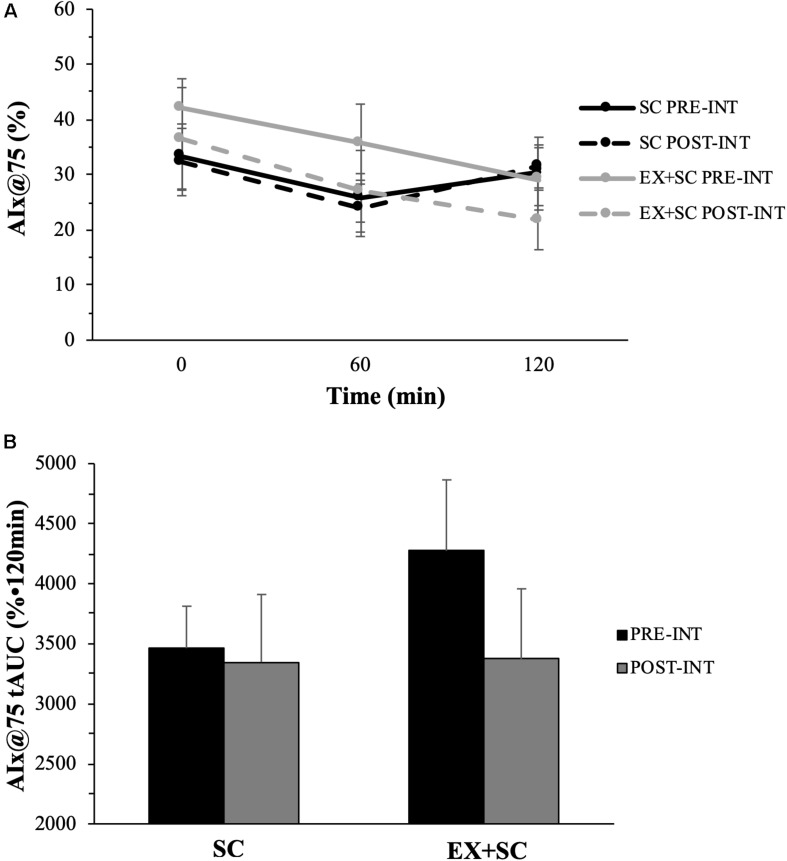
The effect of standard care (SC) and aerobic exercise combined with standard care (EX + SC) on mixed meal tolerance test augmentation index normalized to a heart rate of 75 beats per minute (AIx@75) curves **(A)** and total area under the curve (tAUC) **(B)**. Data are means ± SEM. There were no statistically significant differences, however, EX + SC had a modest effect size (ES) for decreased fasted (ES = 0.20), 60 min (ES = 0.33), 120 min (ES = 0.58), and tAUC (ES = 0.52) AIx@75 compared to SC.

Systolic BP was not affected during the study, however, SC had a medium effect size for decreased diastolic BP (*P* = 0.26; ES = 0.63) and MAP (*P* = 0.33; ES = 0.52) compared to EX + SC (Table 2). While EX + SC had no statistical effect on arterial stiffness versus SC, exercise did appear to have modest effect sizes for decreasing 60 min (*P* = 0.52; ES = 0.33) and 120 min (*P* = 0.32; ES = 0.58) AIx@75 as well as AIx@75 tAUC (*P* = 0.36; ES = 0.52) compared to SC (Figure 1). Both treatments similarly reduced fasting PP (*P* = 0.05), yet SC had a greater effect than EX + SC on fasted PP (*P* = 0.36; ES = 0.51) as well as fasted (*P* = 0.60; ES = 0.1.05), 60 min (*P* = 0.74; ES = 0.46), and 120 min (*P* = 0.70; ES = 0.23) AP (Table 2).

**TABLE 2:** Effect of standard care (SC) and aerobic exercise combined with standard care (EX + SC) on cardiometabolic health.

	SC		EX + SC		ANOVA (*P*-value)		Effect size
	PRE	Δ	PRE	Δ	*T*	*G* × *T*	Cohen’s *d*
Blood pressure							
HR (bpm)	70.1 ± 4.9	5.1 ± 2.4	85.4 ± 5.4	1.4 ± 2.1	0.06	0.27	0.62
Systolic BP (mmHg)	121.4 ± 8.9	1.1 ± 3.4	131.5 ± 6.5	2.8 ± 4.2	0.49	0.76	0.17
Diastolic BP (mmHg)	66.0 ± 4.7	−2.2 ± 2.4	73.4 ± 4.0	2.2 ± 2.9	0.99	0.26	0.63
MAP (mmHg)*	84.5 ± 5.9	−1.1 ± 2.1	92.8 ± 4.6	2.4 ± 2.9	0.73	0.33	0.52
Pulse wave analysis							
Fasted PP	51.6 ± 3.8	−8.2 ± 2.2	40.0 ± 2.7	−3.0 ± 5.3	0.05	0.70	0.51
60 min PP*	39.7 ± 1.8	2.8 ± 2.4	38.7 ± 2.6	1.0 ± 4.9	0.68	0.50	0.19
120 min PP	46.6 ± 4.0	−0.3 ± 1.9	35.6 ± 1.6	0.6 ± 3.1	0.51	0.85	0.14
Fasted AP	20.3 ± 4.7	−5.3 ± 3.5	15.9 ± 1.8	−1.9 ± 5.4	0.28	0.60	1.05
60 min AP*	19.4 ± 5.6	−8.3 ± 6.5	13.2 ± 2.5	−0.7 ± 6.4	0.17	0.74	0.46
120 min AP	16.7 ± 3.1	−3.1 ± 2.9	9.8 ± 1.6	−1.6 ± 2.4	0.24	0.70	0.23
Blood substrates							
Fasting glucose (mg/dL)*	100.0 ± 2.4	−4.2 ± 4.0	105.8 ± 6.4	−0.4 ± 6.5	0.53	0.54	0.26
Fasting insulin (μU/mL)	13.4 ± 2.9	−1.8 ± 2.6	8.5 ± 1.8	1.9 ± 1.8	0.97	0.26	0.63
HDL cholesterol (mg/dL)	40.9 ± 3.8	1.9 ± 4.9	53.9 ± 7.0	0.5 ± 4.3	0.73	0.83	0.12
LDL cholesterol (mg/dL)	139.6 ± 7.1	−6.4 ± 17.4	146.1 ± 12.8	2.2 ± 10.1	0.84	0.67	0.23
Total cholesterol (mg/dL)	200.7 ± 7.3	−5.3 ± 20.3	219.0 ± 19.4	4.8 ± 17.6	0.99	0.71	0.20
Triglycerides (mg/dL)	122.9 ± 30.2	−4.6 ± 29.2	114.4 ± 16.3	−4.0 ± 28.6	0.84	0.99	0.01
Inflammation							
hs-CRP (μg/ml)*	4.95 ± 1.27	0.87 ± 0.57	4.54 ± 1.26	−0.16 ± 0.55	0.14	0.31	0.69
CK18 (U/L)	189.3 ± 52.1	22.0 ± 24.8	191.6 ± 34.5	−5.2 ± 19.0	0.47	0.05	3.05

**Data are means ± SEM. There were no baseline differences between groups for any variable. ^∗^Non-normally distributed data are presented in raw version for ease of interpretation. Fasted and 120 min pulse pressure were covaried for baseline values due to differences between groups. Change from pre- to post-intervention (Δ*)*; body mass index (BMI); fat free mass (FFM); waist circumference (WC); heart rate (HR); blood pressure (BP); mean arterial pressure (MAP); pulse pressure (PP); augmentation pressure (AP); high density lipoproteins (HDL); low density lipoproteins (LDL); high sensitivity c-reactive protein (hs-CRP); cytokeratin 18 (CK18); time (T; pre vs. post-intervention); group × time (G × T; SC vs. EX + SC)*.*

### Blood Substrates and Inflammation

SC and EX + SC had no effect on altering TG, HDL, total cholesterol, LDL, or fasting glucose, although SC had medium effect sizes on fasting insulin (*P* = 0.26; ES = 0.63) compared to EX + SC (Table 2). EX + SC had a medium effect size for decreased hs-CRP (*P* = 0.31; ES = 0.69) compared to SC, and EX + SC decreased CK18 compared to an increase with SC (*P* = 0.05; ES = 3.05) (Table 2).

### Insulin Sensitivity and Metabolic Flexibility

EX + SC had a small effect size for increased whole-body insulin sensitivity compared with SC (EX + SC 2.2 ± 1.8 vs. SC 1.36 ± 0.92, *P* = 0.20, ES = 0.33). EX + SC increased metabolic flexibility compared to a lowered effect with SC (EX + SC 0.01 ± 0.01 vs. SC −0.06 ± 0.02, *P* = 0.01, ES = 1.55).

### Quality of Life

EX + SC significantly improved domains of QoL including symptoms (*P* = 0.03), emotions (*P* = 0.03), and social interaction (*P* = 0.05) as well as total score (*P* = 0.03) (Table 3). EX + SC also improved activity/mobility (*P* = 0.02) as well as total score compared to SC (*P* = 0.02) (Table 3).

**TABLE 3:** Effect of standard care (SC) and aerobic exercise combined with standard care (EX + SC) on weight related quality of life.

	SC		EX + SC		Mann–Whitney *U* Test
	PRE	Δ	PRE	Δ	
Symptoms	61.4 ± 7.1	3.1 ± 3.6	64.2 ± 7.5	13.7 ± 4.2^† †^	0.10
Sexual life	58.2 ± 7.4	−2.0 ± 3.3	57.1 ± 9.4	14.2 ± 7.0	0.07
Activity/mobility	71.7 ± 8.9	−3.6 ± 2.4	64.6 ± 8.7	10.4 ± 4.3	0.02
Hygiene/clothing	71.2 ± 6.2	−1.4 ± 2.9	74.3 ± 3.2	1.2 ± 4.1	0.38
Emotions	50.6 ± 5.8	3.0 ± 2.1	49.9 ± 4.5	13.9 ± 5.0^† †^	0.13
Social interactions	68.2 ± 6.7	−1.2 ± 4.2	58.9 ± 6.7	12.0 ± 5.2^† †^	0.07
Total score	62.9 ± 5.4	0.1 ± 2.2	60.2 ± 5.5	11.9 ± 4.0^† †^	0.02

**Data are means ± SEM. Change from pre- to post-intervention (Δ****).****Pre- to post-intervention differences were determined by Wilcoxon Signed Rank test: ^† †^Significant (P < 0.05). Mann–Whitney U Test was used to compare Δ differences*.*

### Surgical Outcomes

EX + SC had a shorter length of hospital stay compared to SC (41.32 ± 4.4 vs. 56.7 ± 5.7 h; *P* = 0.05, ES = 1.38). There was no difference between treatments in length of surgery (SC 2.1 ± 0.3 vs. EX + SC 2.1 ± 0.3 h; *P* = 0.96, ES = 0.03).

### Correlations

**FIGURE 2:**
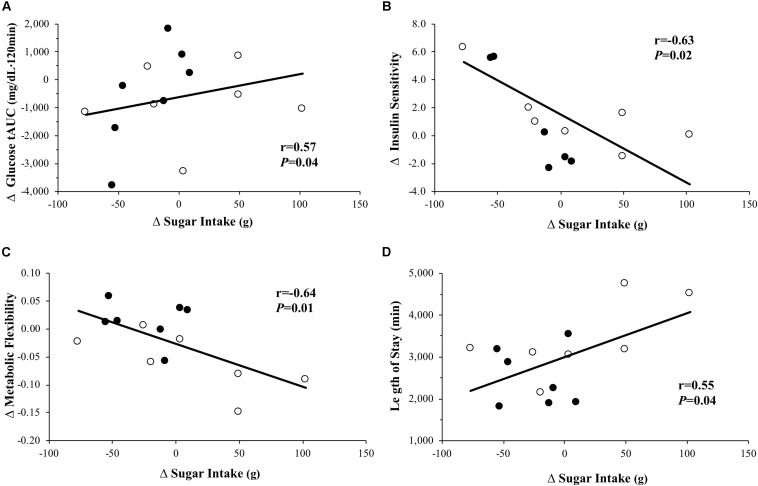
The decrease in sugar intake was associated with a decrease in glucose tolerance **(A)**, increase in whole-body insulin sensitivity **(B)**, and increase metabolic flexibility **(C)** pre- to post-intervention as well as a shorter length of hospital stay **(D)**. Change, Δ; Open circles, standard care (SC); closed circles, aerobic exercise combined with standard care (EX + SC).

A decrease in AIx@75 tAUC correlated with increased whole-body insulin sensitivity (*r* = −0.59, *P* = 0.03) and metabolic flexibility (*r* = −0.60, *P* = 0.03) as well as reduced glucose tAUC (*r* = 0.57, *P* = 0.04). The decrease in AIx@75 tAUC (*r* = 0.65, *P* = 0.02) and increase in whole-body insulin sensitivity (*r* = −0.66, *P* = 0.01) was associated with a reduction in TG. The decrease in caloric intake was associated with an increase in metabolic flexibility (*r* = −0.60, *P* = 0.03) and increased percent of total time in MVPA (*r* = −0.68, *P* = 0.02). The decrease in sugar intake after treatment was correlated with increased whole-body insulin sensitivity (Figure 2; *r* = −0.63, *P* = 0.02) and metabolic flexibility (Figure 2; *r* = −0.64, *P* = 0.01) as well as a shorter length of hospital stay (Figure 2; *r* = 0.55, *P* = 0.04). The increase in number of sedentary bouts was linked to increased fasting glucose (*r* = 0.68, *P* = 0.02).

## Discussion

The main findings highlight that pre-operative EX + SC had no statistically significant effect on lowering fasting and post-prandial arterial stiffness, although there were medium effect sizes. Moreover, reductions in AIx@75 tAUC were significantly correlated with improved whole-body insulin sensitivity, metabolic flexibility, glucose tolerance as well as triglycerides. Together, this suggests the reductions in AIx@75 tAUC after EX + SC are of potential physiologic relevance. Prior work showed that 7 weeks of aerobic exercise combined with a LCD elicited a significantly greater reduction in fasting arterial stiffness when measured using pulse wave velocity than a LCD alone in morbidly obese adults (Nordstrand et al., 2013). Our findings somewhat support this prior work as EX + SC had a medium effect size for lowering arterial stiffness as measured by AIx@75 in the post-prandial state compared to SC (Figure 1). This finding may be clinically meaningful as, a 10% decrease in AIx@75 is associated with a relative risk reduction of 1.38 for all-cause mortality (Vlachopoulos et al., 2010). We did not design this study to understand how EX + SC decreased arterial stiffness, but we speculate it could be either due to insulin-mediated vasorelaxation (Greenfield et al., 2007) or decreased vasoconstriction (Muniyappa et al., 2007). Interestingly, our findings suggest that whole-body insulin sensitivity was related to decreased post-prandial AIx@75. This aligns with our findings that dietary sugar intake after treatment was related to improved glucose tolerance and whole-body insulin sensitivity. Given that dietary sugar consumption is known to induce oxidative stress and impair vasculature function, it would be reasonable to expect that lowering dietary sugar would contribute to favorable cardiometabolic health after treatment (Randle et al., 1963; Kelley and Mandarino, 2000). This also provides insight as to why SC may not have improved post-prandial arterial stiffness, given that SC increased dietary sugar intake over the study. Interestingly, we observed that lower dietary sugar intake post-treatment was associated with decreased length of hospital stay (Figure 2), and EX + SC had a significantly shorter length of hospital stay than SC. This suggests a complex interaction of arterial stiffness and whole-body insulin sensitivity as well as dietary sugar intake with surgical outcomes.

Inflammation at the time of bariatric surgery is a predictor of surgical stress (Fernandez et al., 2004; Steinbrook, 2004; Malin et al., 2017). hs-CRP is a non-specific marker of low-grade systemic inflammation that is a predictor for cardiovascular disease risk (Choi et al., 2013). Targeting hs-CRP with pre-operative EX + SC is clinically meaningful as hs-CRP drastically increases in response to surgical stress and takes >7 days post-operatively to return to baseline (Warschkow et al., 2012). hs-CRP decreased 3.5% following EX + SC compared with a 17.6% increase with SC. Although not statistically significant, the medium effect size is not surprising as others have reported that aerobic exercise training alone (Donges et al., 2010) or combined with dietary interventions (Wegge et al., 2004; Jae et al., 2006) reduce hs-CRP in obese adults. The reason SC elicited a 17.6% increase in hs-CRP is outside the scope of the present trial. However, SC had an increase in absolute sugar intake over the study, and prior work has showed that a higher glycemic load diet induces increased IL-6 secretion from adipocytes which promotes increased CRP synthesis in the liver (Wellen and Hotamisligil, 2005; Huffman et al., 2007). In contrast, CK18 had a robust lowering effect to our EX + SC treatment. CK18 is a biomarker of hepatocyte apoptosis that predicts and correlates to the development of hepatic inflammation, fibrosis, as well as non-alcoholic steatohepatitis (Maher et al., 2015). EX + SC decreased CK18 by 23.6% compared to an 11.6% increase with SC. It is established that diet (Colles et al., 2006; Edholm et al., 2011) and exercise (Fealy et al., 2012; Wu et al., 2017) improve liver function, but limited work has evaluated the effect of exercise and/or diet on CK18. Our findings are consistent with prior work showing that 7 days of treadmill walking at ∼85% of HR_*max*_ caused a significant decrease in CK18 in obese adults with non-alcoholic fatty liver disease independent of weight loss (Fealy et al., 2012). Lower CK18 may improve with EX + SC through decreased oxidative stress, hepatic triglyceride stores, or Mallory bodies (Schweizer et al., 2006; Maher et al., 2015). Why SC in contrast had increased plasma CK18 is unclear given the metabolic control prior to testing. But the findings are consistent with lack of physical activity promoting inflammation. Together, while there are several other inflammatory markers (i.e., MCP-1, TNF-α, etc.) that should be considered, this improved liver inflammation observed in the present study prior to bariatric surgery may be important for hepatic function and surgical outcomes (Goh et al., 2018).

A low QoL prior to surgery is associated with worse physiological surgical outcomes and post-operative QoL (Levett and Grimmett, 2019). It is established that dietary restriction and/or exercise interventions are effective at improving QoL (Carson et al., 2014; Kolotkin and Andersen, 2017), yet few have evaluated the effect of pre-operative lifestyle interventions on QoL in bariatric patients. Baillot et al. (2013) recently showed that 12 weeks of pre-surgical aerobic and resistance training prior to bariatric surgery improved QoL domains including emotions, social interactions, sexual life, and overall QoL as determined by the Laval questionnaire. However, this prior work (Baillot et al., 2013) did not evaluate the effect of improvements in QoL as it relates to cardiometabolic health at the time of surgery or surgical outcomes presented herein. Interestingly, we found no relationship between changes in pre-operative QoL and surgical outcomes in the present study. It is possible we observed no relationship because of the length of our intervention or our sample of bariatric patients having a higher baseline QoL than most patients receiving bariatric surgery (Therrien et al., 2011; Biron et al., 2018). Nevertheless, a major finding of the present study is that EX + SC improved the activity/mobility domain of weight-related QoL compared to SC, thus indicating that EX + SC participants increased their perception of being more physically active/mobile. When using accelerometry, however, individuals undergoing EX + SC increased their sedentary behavior by 3% while there was no change with SC. These findings align with prior work suggesting that individuals initiating an exercise intervention compensate for the increased exercise energy expenditure by becoming more sedentary (Melanson, 2017). This suggests that the increased perception of activity/mobility QoL may be more related to exercise and/or aerobic fitness rather than non-exercise activity. Regardless, this change in non-exercise behavior could be important for understanding cardiometabolic benefit prior to surgery. Specifically, we observed increased sedentary behavior was directly associated with higher fasting glucose concentrations. Additionally, exercise energy expenditure induced weight loss may have been negated by shifts toward more sedentary behavior. In fact, a 3% increase in sedentary time can equal up to 43 min per day, which is similar to the average duration of EX + SC exercise sessions (∼37 min/session). In addition, while we did not see weight loss differences between groups, we observed that increased time spent in MVPA was associated with decreased caloric intake. Herein, individuals undergoing EX + SC complied better to the pre-operative LCD by decreasing energy intake by 747 kcal compared to the 95 kcal decrease seen with SC. This is an interesting finding since we did not control for dietary intake aside from providing the pre-operative standard medical care dietary recommendations. We cannot exclude the possibility that the increased MVPA observed in this study with EX + SC promoted favorable changes in appetite regulation that contributed to lower food intake while on a LCD, however, the changes in exercise/diet behavior with EX + SC may have also occurred due to prompting (Michie et al., 2013). Indeed, electronic reminders with interventions have been reported to result in greater adherence and compliance (Fenerty et al., 2012; Vervloet et al., 2012). Thus, we may have promoted greater adherence to the pre-operative diet by remaining in contact with EX + SC participants about exercise compliance, albeit self-reported food intake presents challenges including misreporting of dietary intake and/or alterations in food choices due to heightened awareness of food consumption (Hedrick et al., 2012; Myhre et al., 2018). These data suggest that adding aerobic exercise to SC provides positive impact on QoL prior to bariatric surgery for individuals.

A somewhat surprising observation was that neither treatment had statistically significant improvements or medium to large effect sizes in blood pressure, triglycerides, cholesterol, fasting glucose, or waist circumference in the present study. This could be explained by several reasons. First, this may relate to the fact that clinical values were fairly normal to borderline of clinical cut-points for hyperglycemia, dyslipidemia, and/or hypertension. As a result, it would be fair to anticipate that EX + SC or SC would have little effect. Second, the lack of change with EX + SC could be due to our exercise prescription. Participants in the EX + SC completed ∼4.3 moderate intensity exercise sessions per week and expended 1,064 kcal/week. This is comparable to the STRRIDE trial, whereby it was reported the low amount/moderate intensity group (i.e., 1,220 kcal/week) had no change in similar cardiometabolic risk factors. Yet, high amount/vigorous intensity exercise (i.e., 2,014 kcal/week) lowered blood pressure and raised HDL in overweight and obese adults (Slentz et al., 2004; Johnson et al., 2007; Slentz et al., 2007), suggesting that exercise intensity or higher energy expenditure may relate to greater improvements in some cardiometabolic risk factors. Lastly, EX + SC or SC had minimal impact on weight loss/body fat reductions in the current study. Perhaps longer lifestyle treatments are required to see greater change in body composition that drive change in these cardiometabolic risk factors. Additional well-controlled studies in patients with clinical cases of cardiovascular disease are needed to determine relevance to surgery outcomes and to identify optimal exercise “doses” to maximize health and well-being prior to and after bariatric surgery.

The present study has limitations that require acknowledgment. This study had a small sample size (*n* = 14), and therefore findings may not be generalizable to the bariatric population as a whole. For instance, race/ethnicity (Wee et al., 2016), sex (Duval et al., 2006; Wee et al., 2016), socioeconomic status (Kim and Park, 2015), education (Kim and Park, 2015), and age (Netuveli et al., 2006) are all known determinants of QoL. Further, due to our small sample size we are not able to determine the effect of SC versus EX + SC on cardiometabolic health, QoL, or surgical outcomes based on surgery type (i.e., RYGB and SG) or sex. Associations do not equal causation, therefore trials with larger sample sizes are needed to better understand the contribution of physiological, psychological, and/or behavioral factors on cardiometabolic and surgical outcomes in bariatric patients undergoing pre-operative lifestyle interventions. We did not have an EX only group, so we cannot be certain of the independent effects of EX without SC on cardiometabolic risk factors or QoL. Although pulse wave velocity is the gold standard for determining arterial stiffness (Cecelja and Chowienczyk, 2012), we were unable to capture this measure in our patients due to the adiposity around the neck region. AIx@75 is highly correlated to pulse wave velocity (Cecelja and Chowienczyk, 2012) and a reasonable surrogate measure of arterial stiffness in this population.

In conclusion, EX + SC had favorable cardiometabolic health effects as well as improved weight related QoL compared to SC in patients with obesity prior to bariatric surgery. Exercise had a greater effect for increased adherence to pre-operative dietary recommendations, and the decrease in sugar intake pre-operatively was associated with a shorter length of hospital stay. Collectively, these findings suggest that pre-operative EX + SC may be important for cardiometabolic health, quality of life, and surgical outcomes in the bariatric patient.

## Data Availability Statement

The raw data supporting the conclusions of this article will be made available by the authors, without undue reservation, to any qualified researcher.

## Ethics Statement

The studies involving human participants were reviewed and approved by University of Virginia’s Institutional Review Board. The patients/participants provided their written informed consent to participate in this study.

## Author Contributions

SM conceptualized the research. NG, NE, and ER were responsible for subject recruitment and retention. NG, NE, MK, and PH were primarily responsible for data collection and management. NG, SK, AW, PH, and SM analyzed and interpreted the data. NG and SM were primarily responsible for writing the manuscript. NG, NE, MK, ER, SK, AW, PH, and SM reviewed and edited the manuscript. All authors contributed to the article and approved the submitted version.

## Conflict of Interest

The authors declare that the research was conducted in the absence of any commercial or financial relationships that could be construed as a potential conflict of interest.
